# Functional iron deficiency anemia in patients with cancer

**DOI:** 10.1007/s44313-024-00030-w

**Published:** 2024-08-07

**Authors:** Jeong Suk Koh, Ik-Chan Song

**Affiliations:** Division of Hemato-Oncology, Department of Internal Medicine, Chungnam National University Hospital, 282 Munwha-Ro, Jung-Gu, Daejeon, 35015 South Korea

**Keywords:** Functional iron deficincy, Anemia, Cancer, Hepcidin

## Abstract

Anemia is frequently observed in patients with cancer owing to anticancer chemotherapy, radiation therapy, and inflammatory responses. This often leads to functional iron deficiency, characterized by adequate iron stores but impaired use of iron for red blood cell production. This condition, termed functional iron deficiency anemia (IDA), is identified by a ferritin level of 30–500 µg/dL and a transferrin saturation < 50%. Functional iron deficiency often develops with the prolonged use of erythropoiesis-stimulating agents, leading to a diminished response to anemia treatment. Although oral iron supplementation is common, intravenous iron is more effective and recommended in such cases. Recent studies have shown that ferric carboxymaltose (FCM) is effective in treating functional IDA in patients with cancer. However, because of its potential to induce asymptomatic severe phosphate deficiency, it is important to closely monitor phosphate levels in patients receiving FCM.

## Introduction

Iron deficiency anemia (IDA) is the most prevalent nutritional deficiency globally, affecting approximately one in six individuals [1, 2]. It is particularly common in low- and middle-income countries, where it constitutes a leading cause of disability, with a prevalence of approximately 30%. In South Korea, data from the 2010 National Health and Nutrition Examination Survey reported IDA prevalence rates of 0.7% in men, 8.0% in women, and 11.5% in women of childbearing age [3–5]. Iron plays an important role in the body by forming hemoglobin (Hb) in red blood cells (RBCs) and myoglobin in muscles. A typical adult contains approximately 4 g of iron, of which half is stored as ferritin in the liver, spleen, and bone marrow, while other half is used by circulating RBCs and muscle tissues [6]. The body lacks an active mechanism for excreting iron, which is passively lost through menstruation and the shedding of mucosal and epidermal cells. Typically, 1–2 mg of iron is lost and absorbed daily through the diet to maintain balance [7]. IDA occurs when the body’s iron loss and demand exceed iron intake.

Iron is chemically versatile and can switch between ferrous (Fe^2+^) and ferric (Fe^3+^) states, which enables its role in oxygen-binding molecules, such as Hb and myoglobin, as well as in cytochromes and various enzymes [8]. However, this reactivity allows iron to form free radicals, that can damage cell membranes, proteins, and DNA. To reduce this risk, iron in the body is tightly bound to proteins such as transferrin in the plasma and stored within cells as ferritin or hemosiderin [6, 8].

### Diagnosing IDA and evaluating its causes

The diagnosis of IDA in patients with microcytic anemia involves measuring parameters such as serum levels of iron and ferritin, total iron-binding capacity (TIBC), and transferrin saturation (TSAT) [9, 10]. TIBC measures the capacity of transferrin to bind to iron in the blood, providing an indirect measure of the iron available to the body. The TSAT was calculated using the following formula: TSAT = (serum iron level × 100) / TIBC. In typical IDA cases, iron, ferritin, and TSAT levels are low, whereas TIBC levels are elevated (Table 1). However, ferritin levels may appear normal or high in the presence of infections or inflammatory diseases such as cancer.

**Table 1:** Differential diagnosis of IDA, anemia of inflammation and functional IDA

**Tests**	**Iron deficiency anemia**	**Anemia of Inflammation**	**Functional IDA**
Blood Smear	Microcytic and hypochromic RBCs	Normal, Microcytic and hypochromic RBCs	Normal
Serum Iron (µg/dL)	< 30	< 50	Variable
TIBC	> 360	< 300	Variable
Transferrin Saturation	< 10%	10–20%	< 50%
Ferritin (µg/dL)	< 15	30–200	30–500

*TIBC* Total Iron binding capacity

Beyond diagnosis, identifying and addressing the underlying causes of IDA is crucial (Table 2). In adults, chronic occult blood loss is often owing to gastrointestinal (GI) issues [11]. Menstrual hypermenorrhea is frequently identified in women of childbearing age, whereas GI blood loss because of malignant tumors is more common in men and postmenopausal women. Diagnostic procedures may include stool occult blood tests and endoscopic examinations such as gastroscopy and colonoscopy. If bleeding in the small intestine is suspected and is not visible via these methods, capsule endoscopy or computed tomography of the abdomen might be considered.

**Table 2:** Cause of iron deficiency anemia

**Increased iron loss**	**Inadequate iron absorption**	**Increased iron demand**
Gastrointestinal blood loss: epistaxis, varices, gastritis, ulcer, tumor, Meckel’s diverticulum, vascular malformation, inflammatory bowel disease, diverticulosis, hemorrhoids Genitourinary blood loss: menorrhagia, cancer, chronic infection Others: trauma, excessive phlebotomy, cupping	Gastrectomy (partial or total) Bariatric sleeve gastrectomy Inflammatory bowel disease *Helicobacter pylori* infection Antacid, H_2_-blocker, proton-pump inhibitor therapy or high gastric pH Excessive dietary bran, tannin, phytates, or starch	Rapid growth (adolescence) Pregnancy Erythropoiesis stimulating agents use

In regions such as Korea and Japan, where gastric cancer is prevalent, gastrectomy is a common procedure that has recently been used to treat obesity using procedures such as sleeve gastrectomy [12, 13]. These surgeries can contribute to IDA by bypassing the areas of the intestine critical for iron absorption, reducing gastric acid secretion, and accelerating gastric emptying [14]. The onset of IDA after gastrectomy typically occurs within 5–10 years, depending on the patient’s age and nutritional status. Therefore, regular monitoring of iron and vitamin B_12_ levels is essential in these patients.

### What is functional IDA?

**Fig. 1:**
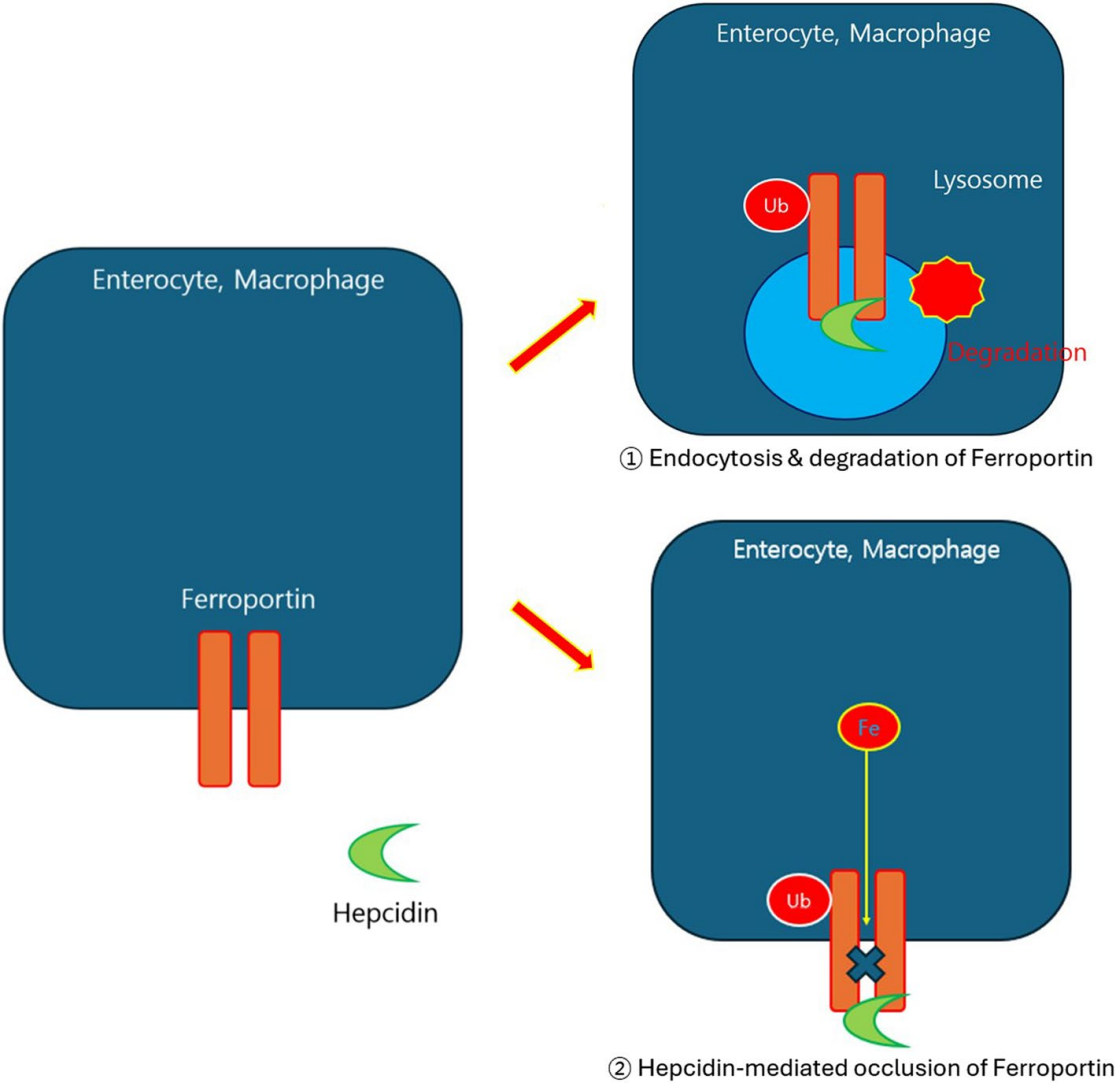
Mechanism by which hepcidin inhibits iron export through ferroportin

Functional IDA is characterized by adequate iron storage; however, the bioavailability of the iron required for RBC production is poor [15, 16]. This often results from the blockade of iron use by hepcidin, a condition that can occur during inflammatory conditions or in older adults [17, 18]. Hepcidin, which is produced in the liver, was discovered in January 1998 in the SWISS-PROT database as a protein (P81172) and was initially thought to have bactericidal properties. However, subsequent research has revealed that hepcidin primarily regulates iron levels [19]. During inflammation or infection, hepcidin production increases owing to interleukin-6, which interferes with iron absorption by blocking ferroportin in the GI tract, decreasing serum levels of iron by blocking ferroportin in macrophages where iron is stored, thereby preventing iron release [20, 21]. Hepcidin inhibits iron export via ferroportin through two mechanisms (Fig. 1). First, it binds to ferroportin, causing its ubiquitination and subsequent endocytosis and degradation in lysosomes [20]. Second, hepcidin directly occludes the central cavity of ferroportin, preventing iron transport [21]. For this binding, iron is required in the central cavity of ferroportin, and the binding affinity of ferroportin for hepcidin increases 80-fold in the presence of iron [22].

The thresholds for diagnosing functional IDA vary depending on the specific disease. For example, in patients with heart failure, functional IDA is defined as ferritin levels < 100 µg/L or < 300 µg/L with a TSAT of < 20%. In patients with chronic kidney disease, it is delineated as ferritin levels not exceeding 500 µg and a TSAT of ≤ 30% [23, 24]. For patients in the postoperative phase, functional IDA is characterized by ferritin levels < 100 µg and/or a TSAT < 20% [25]. In patients with cancer, functional IDA is identified with ferritin levels ranging 30–500 µg/dL and a TSAT < 50% [15]. Furthermore, in patients with cancer, possible functional IDA is defined by a TSAT of < 50% and a ferritin level between 500 and 800 ng/mL.

### Anemia in patients with cancer

Anemia in patients with cancer can be caused by various factors, including blood loss, renal insufficiency, chronic inflammation, and the cancer itself, all of which can exacerbate the condition [26]. Cancer cells may directly impair erythropoiesis by sequestering iron, decreasing RBC production, and reducing RBC survival via cytokine production [27]. In addition, bone marrow suppression can occur when patients receive chemotherapeutic agents or radiotherapy targeting the skeleton, resulting in anemia. Therefore, evaluating the cause of anemia in patients with cancer requires the consideration of multiple factors.

Treatment options for anemia in patients with cancer include blood transfusions, erythropoiesis-stimulating agents (ESAs), and iron supplements [27]. Blood transfusions are effective for rapidly correcting anemia; however, it is important to monitor for transfusion-associated circulatory overload and iron overload in patients receiving frequent transfusions [28, 29].

ESA therapy does not provide an immediate increase in Hb as transfusions do, but it does gradually improve anemia over 2–3 weeks, with an Hb response observed in approximately 65% of patients [30]. In a randomized placebo-controlled study, epoetin alfa significantly increased hemoglobin levels (2.2 vs. 0.5 g/dL; *P* < 0.001) and reduced transfusion requirements (24.7% vs. 39.5%; *P* = 0.0057) in patients receiving chemotherapy for anemia [31]. Another randomized phase III study found that patients with lung cancer and Hb levels ≤ 11 g/dL receiving chemotherapy and darbepoetin alfa required fewer transfusions (27% vs. 52%; 95% confidence interval [CI], 14–36%; *P* < 0.001) compared to those receiving placebo [32]. The ability of ESAs to reduce transfusions was also highlighted in a Cochrane review involving 20,102 patients with cancer undergoing treatment with ESAs [33], which showed a reduced relative risk (RR) for transfusion in patients treated with ESAs (RR, 0.65; 95% CI, 0.62–0.68). A meta-analysis assessing the efficacy of darbepoetin alfa initiated at Hb ≤ 10 g/dL in anemic patients with cancer revealed that more patients receiving darbepoetin alfa achieved a Hb increase ≥ 1 g/dL (fixed-effects HR, 2.07; 95% CI, 1.62–2.63) or ≥ 2 g/dL (HR, 2.91; 95% CI, 2.09–4.06) compared to those on placebo [34], with fewer transfusions needed (HR, 0.58; 95% CI, 0.44–0.77).

However, ESAs are associated with risks, including increased thrombotic events, potentially decreased survival, and accelerated tumor progression [35–37]. It is critical to discuss the risks of ESA therapy with patients, including the potential for tumor growth, death, blood clots, and hypertension. Continuous monitoring of the iron profile is also necessary when administering ESAs because functional IDA may occur.

### Treatment of functional IDA

In cases of absolute IDA, both oral and parenteral iron can be effectively used. However, for functional IDA, parenteral iron therapy is preferred because of the poor absorption of oral iron from the GI tract, which is hindered by hepcidin [16]. In addition, intravenous (IV) iron, rather than oral iron, has been shown to improve Hb response in patients with cancer treated with ESAs [38, 39]. A randomized study that compared IV iron to oral iron in 502 anemic patients with cancer revealed that the Hb response was notably better in the IV iron group, particularly for those who received ≥ 80% of the planned dose and had a TSAT < 20%, whereas the response was reduced and the time to response was longer in patients with a TSAT of 20–50% [40].

Recent studies have explored high-dose IV iron administration using ferric carboxymaltose (FCM) in various medical conditions. An observational study of 364 patients with cancer who had at least one follow-up Hb measurement reported a median Hb increase of 1.4 g/dL with FCM alone and 1.6 g/dL when combined with ESAs [41]. Patients with Hb < 11 g/dL and ferritin < 500 µg/mL particularly benefited from FCM, and those with ferritin > 500 ng/mL similarly experienced benefits when they had lower TSAT. Moreover, a prospective observational study in France observed that 1000 mg FCM increased Hb by 1.3 g/dL within 3 months in patients with solid or hematologic cancers and by 1.4 g/dL when combined with ESA [42]. A retrospective study of 303 patients in Belgium found that Hb increased by 0.5 g/dL with FCM, with a more pronounced response in patients with low baseline levels of ferritin (< 100 µg/L) [43].

Furthermore, a randomized study in preoperative patients with colon cancer (IVICA trial) compared oral iron and FCM, finding that IV iron did not reduce blood transfusions but was more effective than oral iron at treating preoperative anemia, with Hb increasing by 1.55 g/dL with FCM vs. 0.5 g/dL with oral iron (*P* < 0.001) [44]. In addition, a randomized trial that compared FCM to standard care in patients with postoperative functional IDA (defined as Hb 7–12 g/dL and ferritin ≤ 100 µg/L or TSAT ≤ 20%) demonstrated that FCM significantly reduced the number of postoperative transfusions in the FCM arm [25].

However, the FCM results were not uniform or positive. The FIT study, a multicenter randomized trial, compared FCM with oral iron supplementation in patients with colorectal cancer and IDA undergoing surgery. There was no significant difference in the proportion of patients with normal Hb levels between the FCM and oral iron groups [45]. In addition, the PREVENTT trial, another randomized controlled study, reported that preoperative administration of FCM to patients with anemia undergoing major open elective abdominal surgery did not effectively reduce the number of transfusions compared to placebo [46].

The common adverse effects of IV iron therapy include hypotension, hypertension, nausea, vomiting, diarrhea, pruritus, headache, and dizziness [47]. Specifically, the use of FCM often leads to hypophosphatemia, although this condition is rarely symptomatic or clinically problematic [48, 49].

## Conclusion

Functional IDA can easily be overlooked in patients with cancer. Despite some controversy, IV iron therapy, particularly FCM, remains a viable option for correcting functional IDA, although careful monitoring is essential. Recent research has broadened the potential applications of FCM for functional IDA in various medical conditions, necessitating the meticulous monitoring of iron status to effectively manage this condition.

## Data Availability

This declaration is not applicable because this paper is a review article that searches for and organizes existing literature.

## Data availability

No datasets were generated or analysed during the current study.
